# One image is worth more than a thousand words: producing an atlas of medical signs for teaching clinical and forensic toxicology

**DOI:** 10.1080/20961790.2022.2059837

**Published:** 2022-05-03

**Authors:** Ricardo Jorge Dinis-Oliveira

**Affiliations:** a TOXRUN – Toxicology Research Unit, University Institute of Health Sciences, Advanced Polytechnic and University Cooperative (CESPU), CRL, Gandra, Portugal; b Department of Public Health and Forensic Sciences, and Medical Education, Faculty of Medicine, University of Porto, Porto, Portugal; c UCIBIO-REQUIMTE, Laboratory of Toxicology, Department of Biological Sciences, Faculty of Pharmacy, University of Porto, Porto, Portugal

**Keywords:** Forensic sciencesForensic sciences, clinical and forensic toxicologyclinical and forensic toxicology, teachingteaching, learninglearning, signs of exposuresigns of exposure, pre-analytical phasepre-analytical phase

## Abstract

Clinical and forensic toxicology are critically involved in the acquisition of basic skills to correctly suspect intoxication, collect biological and non-biological materials for toxicological analysis, comprehend the complexities inherent to laboratory activity, and understand the fundamentals of toxicokinetics and toxicodynamics that underlie the interpretation of results. This works presents a pedagogical innovation proposal for the teaching of clinical and forensic toxicology based on a compilation of more than 3 000 cases where the image was fulcra for suspicion. The experience in this article follows the model practiced in bachelors, masters, and PhD degrees, as well as in other continuing training courses, where we are teaching toxicology for more than 15 years. All these levels of education are considered fundamental to the sound development of this science. This approach aims also to offer strength to the intervention of the true toxicologist in all the toxicological phases, besides the classic analytical chemistry. Indeed, it is impossible to provide effective clinical and forensic toxicological interpretations without a proper and broad education, and not thinking exclusively in terms of laboratory techniques. In the future, it will be interesting to evaluate knowledge retention and to propose a database of videos of signs related to intoxications.

## Key points

A pedagogical innovation proposal for the teaching of forensic and clinical toxicology is presented.

A universal and never-ending atlas of phtotographs related to signs of intoxications have been compile.

Offering to our students an integrated teaching of clinical and forensic toxicology is crucial since both are grounded in analogous toxicological principles and are mutually dependent.

## Introduction

Toxicology is arguably one of the oldest sciences in the service of humanity, as it has existed since the first humans sought to identify safe plants and animals for feeding. Since then, the knowledge of the toxic and curative properties of plants, animals, and minerals has shaped civilization for millennia. Thus, toxicology evolved steadily throughout the history, existing today as an independent scientific entity with its own dedicated researchers and methodologies. Knowledge of toxicology is becoming increasingly relevant in multidisciplinary scientific intervention. Moreover, toxicology was (and still is) fulcra for the development of other sciences, including medicine, and is now recognised as an integrative area of chemistry, biochemistry, biology, genetics, immunology, pharmacology, pathology, histology, and many other disciplines.

For the growth of toxicology, teaching evolution is crucial to prepare students and future experts to work correctly and appropriately, under the law, when confronted in their professional life with issues of clinical and forensic nature. This thereby contributes to the sound administration of justice and adequate treatment of patients. The effectiveness of that process is completely dependent on education and knowledge. However, an evaluation of how we are teaching these subjects is almost non-existent because routine casework is largely separate from academics. Respectable books have been considered hallmarks in toxicology and are key for anyone who is aiming to enter the field [1–6]. Yet, issues related to the suspicion and recognition of signs of intoxication are not described in-depth in textbooks or are typically relegated for second plan, being mostly described in isolated case reports. Indeed, clinical, and especially forensic toxicology have been perceived more as analytical chemistry science-based fields without considering its broad-spectrum application as it was in the origin of toxicology. This means that toxicologists in most countries work in a laboratory and only provide chemical analysis results without participating in clinical decisions or autopsies. They also usually do not deal with the interpretation and integration of chemical results with other clinical and pathological findings. This leads to a tremendous loss of potential since the toxicologist has a much powerful background and should work together with pathologists. I have noticed since the beginning of my relationship with clinical and forensic toxicology that I could offer much more than exclusively doing analytical chemistry analysis. Therefore, it is important that the toxicologist returns to the major focus of the field and participates in all four steps of the toxicological framework: (i) suspicion; (ii) collection of biological and non-biological samples and specific guidelines have been proposed [7–10]; (iii) analytical chemistry analysis; and (iv) interpretation of the results. While the presence of a toxicologist for analytical chemistry analysis is fully recognised for his/her background in pre-analytical steps and instrumental analysis, it is also true that if this is the unique aspect of toxicologist intervention, then he/she should be asked as an analytical chemist and not as a toxicologist. It other words, it is not adequate to called him/her a “toxicologist” by the simple fact that he/she is analysing the xenobiotics and endobiotics involved in intoxication. This controversy and entropy regarding the correct definition of a toxicologist are enhanced by those that only perform analytical work. Attempts to solve this issue have been previously proposed [11,12].

This work presents a pedagogical innovation proposal for the teaching of forensic and clinical toxicology based on the compilation of a database of more than 3 000 cases disclosed by image. This represents an advancement in students active learning in addition to audio and video podcasts, flipped classroom learning, and e-books.

## Objectives of the curricular unit of clinical and forensic toxicology

The primary objective is to immerse students into the world of clinical and forensic toxicology in its various divisions. More specifically, it is aimed to provide basic knowledge on the various areas of expertise of clinical and forensic toxicology in terms of their scope, objectives, skills, and pertinent legislation. Emphasis need to be placed on the signs and symptoms that may lead to suspicion of intoxication, interpretation of the toxicological results, and how this science can and should be integrated with the other areas of clinical and forensic intervention. In particular, at the end of a curricular unit the student should have acquired skills:

- To master the basic theoretical and practical aspects of toxicology, as well as the basic principles of epidemiology and surveillance of poisonings because they can uncover relevant trends of intoxications.
- To understand the disposition of xenobiotics in biological systems, including absorption, distribution, metabolism, and excretion (ADME), and how this contributes to the toxicity of the substance.
- To learn and identify the factors that affect ADME and consequently the pharmacological or toxicological responses, with particular focus on toxicogenetics, toxicogenomics, and species specificities.
- To understand the most important antidotes.
- To understand the adverse effects of xenobiotics such as immediate *versus* delayed effects, reversible *versus* irreversible toxicity, local *versus* systemic toxicity, interactions between xenobiotics, tolerance, idiosyncratic, allergic reactions, genotoxicity, carcinogenicity, and reproductive and developmental toxicology.
- To identify and understand the molecular and cellular mechanisms of action of xenobiotics that are most commonly involved in poisonings: pesticides, metals, volatile gases, psychoactive substances (such as opioids, hallucinogens, depressants of the central nervous system like benzodiazepines, barbiturates, and ethanol, stimulants as amphetamines and cocaine, *Cannabis* derivatives, and inhalants), drugs, and caustics.
- To recognise the analytical methodologies for screening and confirmatory analyses used in toxicological and pharmacological analysis.
- To understand normal organ physiology and its role in organism homeostasis, then interpret the pathology of toxic effects on these organs and the macroscopic and microscopic aspects of pathological processes.
- To understand the concepts of postmortem redistribution.
- To understand the principles of human performance toxicology, distinguish between legal and illegal drug use and understand the laws that regulate driving under the influence of ethanol and psychotropic substances.
- To recognise the signs and symptoms caused by intoxication with different xenobiotics, as they can offer relevant clues to guide the toxicological analysis.
- To explain the reasons for the vulnerability of different organs and physiological systems to the toxic effects of xenobiotics.
- To understand the major principles of selection, collection, preservation, and packaging of different types of samples (biological or physical).
- To recognise the screening analytical methodologies (commonly known as “screening”) and confirmation processes used in toxicological analyses.
- To understand the toxic effects caused by environmental, ecotoxicological or occupational pollutants (such as gases, vapours, aerosols, and particulates) and basic knowledge of preventive measures and regulatory decisions, namely those with relevance to the broad understanding of occupational diseases.
- To recognise the specific aspects of veterinary toxicology and laboratory animal science and the implementation of the Refine, Reduce, Replace (3R) principles, and properly plan an animal experiment according to legislation and ethics.
- To correctly apply statistical methods in toxicology and interpret their meaning.

As a last resort, the student should be able to request a toxicological analysis and prepare its report.

## Clinical and forensic toxicology

To act as a true toxicologist in the clinical and forensic fields, symbiosis between both areas is necessary. The same recognition is advertised by the learning outcomes, expected skills, and competences for core and specialised topics by the Federation of European Toxicologists and European Societies of Toxicology (EUROTOX) [13]. Although there are relevant differences in who receives the toxicological report, with clinical toxicology focusing on the treatment of a patient while forensic toxicology aiming on clarifying judicial and judiciary issues involving a victim of violence (in a broad spectrum), the truth is that the practice of both areas has relevant similarities regarding techniques and, most importantly, are grounded in analogous toxicological principles. Indeed, forensic investigation is a special form of clinical practice and, like all other clinical specialties, largely depends on the correlation of history, signs of exposure, collection of appropriate samples, application of analytical chemistry techniques, and interpretation of toxicological results by the application of clinical judgment to the entire case. The chain of custody represents the major conceptual difference, but clinical practice also accomplishes rigid accreditation rules. Indeed, each specimen is uniquely linked to a patient, typically by name, patient number, and possibly a bar code. The name of the individual who collected the specimen and everyone who handled it are also usually included. In the case of clinical toxicology, it is presumed that no one will tamper with the specimen once it is obtained, while the court does not make such good faith assumptions. Therefore, sample integrity and security are hallmark. One interesting balance of both areas can be found in a previous publication [14]. Because of the similar approaches and since one does not leave without the other, we are currently offering our students an integrated programme of clinical and forensic toxicology.

## Atlas of intoxications

To complete an integrated teaching plan, I have been strengthening the teaching of the clinical and forensic toxicology curricular units since 2008 with a syllabus that is different from those in the past. Particularly, we have been encouraging understanding the toxicokinetics and toxicodynamics of different xenobiotics and the factors that influence them and advocating the use of compiled tables of toxic and lethal doses with the due care. The student needs also to understand that there are no absolute rules for the interpretation of toxicological results because they are not simple and therapeutic, toxic, and lethal concentrations frequently overlap. Therefore, the teaching of clinical and forensic toxicology should include issues related to the signs and symptoms leading to suspicion of intoxication and the correct interpretation of the toxicological findings. Indeed, the good practices in clinical and forensic toxicology, as in most analytical-based fields, consider that detailed pre-analytical information is fundamental for the success of the toxicological process. Specifically, this approach allows for the appropriate selection of biological samples and may also help select the most adequate toxicological analytical techniques to be performed.

This work was developed to describe the signs and symptoms related to acute and chronic poisonings by the most frequent xenobiotics and currently discussed by toxicological groups within the curricular unit. A simple systematic analysis to rule out xenobiotic involvement in a case should be reserved for when clinical examination or autopsy findings do not exclude the possibility of intoxication or when it is intended to clarify if an individual was under the influence of ethanol or other psychotropic substances at the time of death. A targeted toxicological analysis is more likely to be successful when suggestive signs point to a specific xenobiotic or group. However, this demands an extensive and continuous casuistic approach and the intervention of a true toxicologist, which is not possible in those professionals possessing only analytical chemistry skills. To address this, a compilation of case reports related to our routine work over more than 15 years, together with those written in English, German, French, Spanish, and Portuguese indexed in MEDLINE, were searched, and reviewed for specific signs related to xenobiotic exposure. The corresponding authors of relevant articles were contacted to obtain reports of new cases where photographs of signs related to intoxications were obtained. This approach made it possible to consolidate a portfolio/database/atlas of more than 3 000 intoxication-related photographas, which may now be useful in toxicological analysis. Some previous data can be found in specific publications [15–25], and further publications will continue to be prepared to fully characterise intoxications by signs and symptoms. Table 1 is a compilation of the major signs of xenobiotic and endobiotic exposure in living or deceased individuals. Specific physical aspects of the scene were not included in this work. This approach has been subjected to a constant renewal to maintain up-to-date curricular content and teaching and learning methodologies. Interestingly, this compilation demonstrates that no intoxication exhibits pathognomonic signs or symptoms, suggesting that toxicological analysis is inevitably required. This process is now much more focused and undoubtedly more successful. I have also been advocating that these classes should be organised in discussion models by referring to specific cases with a practical application. To achieve these aims, atlas with images related to intoxications have been a powerful help. This current method also aimed to limit the time of some theoretical classes to meet the expectations of a new generation of learners [26]. In addition, my pedagogical models also embrace student participation in real situations, namely in living exams, autopsies, and court trial participation. This experience has shown that teaching and learning are not limited to attending classes, nor to acquiring knowledge through the exhaustive memorisation of details previously selected by the teacher. These syllabuses are then easily forgotten by the student without regular practical use [27]. For students, problem-based learning methodologies using a database of cases allow complex information to be understood and retained more efficiently [28].

**Table 1.** Suggestive signs related to intoxications/exposures to xenobiotics or endobiotics.

Xenobiotic/endobiotic	Suggestive signs
Ethanol	Steatosis or fatty acid liver Micronodular and macronodular hepatic cirrhosis Jaundice Splenomegaly Hepatocellular carcinoma Gastritis Oesophageal varices rupture *Caput medusae* Ascites Coagulative disorders: petechiae, purpura and ecchymosis Xanthelasma Phrynoderma/follicular hyperkeratosis Bitot’s spots Angular cheilitis or perlèche Atrophic glossitis (i.e. magenta cobblestone tongue) Pellagra Acrodermatitis enteropathica Gynecomastia Dilated cardiomyopathy Vomit aspiration Facial flushing Palmar erythema or ‘‘liver palms’’ Spider telangiectasias *Porphyria cutanea tarda* Psoriasis Rhinophyma (‘‘drinker’s nose’’) Dupuytren’s contracture Madelung’s disease Cullen’s sign (i.e. periumbilical ecchymosis) Grey-Turner’s sign (i.e. flank ecchymosis) Pancreatic panniculitis Black hairy tongue (lingua villosa nigra) Digital clubbing Koilonychia or concave or spoon-shaped Muehrcke’s lines or onychorrhexis or apparent leukonychia Terry’s finger and toenails (leukonychia) Red lunulae Onycholysis Foetal alcohol syndrome Sialosis Testis atrophy
Cocaine	Palatal and septal necrosis and perforation due to snorting Nose deformity, loose of supporting and collapse of bone structures involving the midface, the nose, and the upper lip Long little fingernail used to deliver powder to the nostrils Bone perforation in the central part of the orbit roof Bruxism and caries Loss of the cuticles of the proximal nail folds and “parrot’s-beaked” clawing of some nails in “crack” Atrophy of the finger pulps (especially the thumbs) and burns in “crack” Lateral eyebrow thinning in “crack” Necrosis and gangrene after injection Urticarial vasculitis Pyoderma gangrenosum Oesophageal varices rupture Levamisole-induced necrotizing vasculitis Brain haemorrhage Body packers or mules Cardiac hypertrophy Mydriasis
Heroin	Track marks and skin-popping scars after repeated injections Nonhealing and chronic ulcers, and necrotizing fasciitis Miosis “Puffy hand syndrome” and local abscesses Nodules of dark brown deposits Diffuse erythema Hyperpigmentation of the tongue in a smoker Bullous skin lesions (“coma blisters”) Bilateral ptosis if contaminated with botulinum toxin “Foam cone’’ exuding from the mouth and nose
Krokodil	Gray-greenish discolouration of the skin resembling the skin of a crocodile Ulcers and necrosis of the skin Gangrene Jaw osteonecrosis Limbs amputation
*Cannabis*	Congestion or conjunctival hyperaemia
Amphetamines and derivatives	“Methamphetamine mouth” characterised by rampant caries (gingivitis/periodontitis) Body packers Crank-bugs in methamphetamine Brain haemorrhage Bruxism in “ecstasy” Mydriasis
Methanol	Bilateral haemorrhage in the basal ganglia (e.g. putamen, lentiform nuclei, globus pallidus) Necrosis of oesophageal and gastric mucosa Oedema with cerebellar tonsillar hernia
Silver	Generalised argyria characterised by a gray-blueish or metallic diffuse skin pigmentation, which becomes evident predominantly in sun-exposed areas, such as head, neck, and hands Azure lunula: bluish-grayish discolouration of the fingernails, more precisely of the lunula Localised argyria: local silver deposition following skin incisions or percutaneous absorption *via* sweat gland pores Amalgam tattoo Argyrosis: ocular silver deposition
Chemical burns	Sulfuric acid: dark-brownish discolouration of the skin and mucosa. Squeals with retraction and impaired of the movements Hydrofluoric acid: silvery-greyish or blue-greyish necrosis Nitric acid: yellowish discolouration of the oral and mucosa Hydrochloric acid: greyish-black discolouration of the oesophagus, larynx, and spleen Hydrogen sulphide: greenish discolouration of the grey matter and nuclei, greenish discolouration of the hypostasis Sodium hydroxide: necrosis with oedema and intense haemorrhagic congestion, the tissues ranging from white, to sloughy grey or to black Cement exposure: oedema, haemorrhage, necrosis, and erythema of the skin Sulphur mustard: vesicant (blistering) effect
Elemental exposure	Fluorosis: white lines or streaks on the teeth and bones deformities Lead: Burton’s line (blue purplish) Thallium: alopecia, Mee’s lines Arsenic: arsenicosis, hyperkeratosis of soles and palms, Mee’s lines, acneiform or pustular eruptions Selenium: dystrophy of fingernails and patchy loss of hair over scalp Iron: hemochromatosis and a bronze pigmentation of the skin Copper: Kayser-Fleischer rings and sunflower cataracts Mercury: hepatomegaly, orange discolouration of the liver and acrodynia Gold: chrysiasis (i.e. blue-grayish to greyish-purple staining of the skin) Bromide: bromoderma characterised by vegetating ulcer, acneiform rash and pustules
Cyanide	Odour to bitter almonds Cherry pink hypostasis Cherry pink blood Cyanosis Pseudohaematuria/pink discolouration of the urine, serum, cerebrospinal fluid, vitreous humour and pericardiac fluid due to hydroxocobalamin Bright pink discolouration of the epicardium due to hydroxocobalamin
Carbon monoxide	Smoke and soot in the face, nostrils, mouth, larynx, trachea, and principal bronchus Cherry pink hypostasis of the skin and internal organs Cherry pink fingernails Pink discolouration of muscles Pink teeth Fluid and cherry pink blood Intramural haemorrhage with myocardial rupture Differential diagnosis with escharotomy and fasciotomy
Methylene blue	Blue-grayish *livor mortis* Blue-grayish discolouration in multiple organs such as the brain, laryngeal mucosa, pulmonary and coronary arteries, and heart after contact with oxygen
Nitrite and nitrates, and other methemoglobinemia inducing agents	Chocolate discolouration of the dark arterial and venous blood and serum Kidney with dark brown medullae and brown myocardium Differential diagnosis with ochronosis E.g. nitro-glycerine, nitroprusside, amyl nitrite, nitric oxide, sulphonamides, dapsone, phenacetin, phenazopyridine, some local anaesthetics such as prilocaine, topical anaesthetics such as Emla^®^ cream, benzocaine, antimalarial drugs, and poppers
Paraquat	Blue-greenish discolouration of the organs is suggestive of paraquat intoxication due to the dye included in the formulation Pulmonary oedema Pulmonary fibrosis Gastrointestinal necrosis Necrosis and ulceration of the tongue
2,3,7,8-Tetrachlorodibenzo-p-dioxin	Chloracne and skin hyperpigmentation
Other suggestive signs of xenobiotic exposure	Pharmacobezoars Coma blisters

## Conclusion

Teaching and communicating with students are very personal experiences. It is shaped by a teacher’s personality, philosophy, socialisation, and commitment, as well as the environment in which he/she works. The institution can decisively influence the socialisation of a clinical and forensic toxicology teacher. Particularly in the case of forensic toxicology, the “CSI effect” over the last two decades has led to an almost exponential increase in the number of courses and students in all academic degrees interested in these areas. They otherwise would not have gone to university or would, perhaps, have chosen a totally different subject of study [29]. All forensic researchers should be thankful to those TV series for inspiring a new generation of students. However, this boost has also led to inconsistencies in skills and competencies acquired by graduates seeking employment in the field, justifying a need for expert certification.

Since the beginning of 2008, I have intended to reflect on how we are currently teaching clinical and forensic toxicology. Firstly, we introduced peer review in the teaching/learning of clinical and forensic toxicology as a new opportunity for pedagogical innovation that favours an evidence-based teaching and peer experience [30,31]. This approach helped outstandingly evolve and offered me confidence to teach the best practices in these areas. In this work, I have prepared a never-ending atlas by compiling the signs that may suggest and point to a presumable intoxication or exposure. The major concern was to prepare a syllabus that is adapted to the curricular plan and to the student’s future place in the professional field. This would not only prepare him/her for the analytical aspect, but for all fields of toxicology. Therefore, the new syllabus has been enabling and encouraging participatory teaching, as this contributes to a greater perception of students’ skills, allowing for a better use of human resources and inter-individual differences in the classroom.

Despite the crucial importance of signs, symptoms, radiological diagnosis, and circumstantial evidence, nothing can replace a toxicological analysis for qualitatively and/or quantitatively demonstrating which xenobiotic(s) and endobiotic(s) are involved and their concentrations. Indeed, the unequivocally truth is that the dose makes the poison. However, qualitative and quantitative analyses are highly dependent on the continuous improvement and development of new analytical techniques or instruments. Additionally, an in-depth knowledge of the effects of xenobiotics and recognising them through images will greatly improve toxicological expertise.

The respect and dedication that we have given to teaching and our students has allowed us to gain an essential memorandum: modesty regarding the knowledge and capabilities we possess, honesty of our attitudes, and humanity that we offer to those who attend our courses. Our teaching and learning methodologies are no longer characterised by the simple diffusion of information between teachers and students, but rather by practices that encourage the students’ competencies in searching for information, integrating their knowledge, being creative, and appropriately responding to individual cases in daily life. At the end of the curricular unit, the students will obtain the most important role in their toxicological preparation: perfectly synthesising their acquired knowledge and establishing relationships with other scientific areas, such as forensic pathology, forensic anthropology, forensic clinic, law, genetics, and forensic biology.

Finally, it is also important to highlight the importance of routine work and academic cooperation. Worldwide, clinical and forensic toxicology have developed largely in isolation from academics. It is important that the specific skills are established through validated and proven knowledge and not through self-taught processes, which often lead to incorrect practices that can ultimately lead to miscarriages of justice. Madea and Saukko [32] described that forensic medicine will only survive as an academic discipline by concentrating on its scientific duties, not by augmentation of routine casework. The Portuguese reality intends to solve almost all problems by facilitating students’ learning and application of that knowledge to routine casework.

In conclusion, it is impossible to provide effective clinical and forensic toxicological interpretations without a proper and broad education, not thinking exclusively in terms of laboratory techniques. The European Credit Transfer and Accumulation System (ECTS) has a good policy and promotes that “learning outcomes place the emphasis on the results of the learning process for the learner in terms of knowledge, understanding and abilities, rather than on the means the teaching staff employs to obtain those results”. Corroborating these achievements, I expect to go deeper in the future by evaluating the knowledge retention with this model and to develop a database of videos of signs related to intoxications.
